# Targeting KRAS in Non-Small Cell Lung Cancer

**DOI:** 10.3389/fonc.2021.792635

**Published:** 2022-01-10

**Authors:** Elena Corral de la Fuente, Maria Eugenia Olmedo Garcia, Ana Gomez Rueda, Yolanda Lage, Pilar Garrido

**Affiliations:** ^1^ Early Phase Clinical Drug Development in Oncology, South Texas Accelerated Research Therapeutics (START) Madrid-Centro Integral Oncológico Clara Campal (CIOCC), Centro Integral Oncológico Clara Campal, Madrid, Spain; ^2^ Department of Medical Oncology, Ramón y Cajal University Hospital, Madrid, Spain

**Keywords:** targeted therapy, NSCLC, KRAS, immunotherapy, drug resistance, lung cancer

## Abstract

Kirsten Rat Sarcoma viral oncogene homolog (KRAS) is the most frequently altered oncogene in Non-Small Cell Lung Cancer (NSCLC). KRAS mutant tumors constitute a heterogeneous group of diseases, different from other oncogene-derived tumors in terms of biology and response to treatment, which hinders the development of effective drugs against KRAS. Therefore, for decades, despite enormous efforts invested in the development of drugs aimed at inhibiting KRAS or its signaling pathways, KRAS was considered to be undruggable. Recently, the discovery of a new pocket under the effector binding switch II region of KRAS G12C has allowed the development of direct KRAS inhibitors such as sotorasib, the first FDA-approved drug targeting KRAS G12C, or adagrasib, initiating a new exciting era. However, treatment with targeted KRAS G12C inhibitors also leads to resistance, and understanding the possible mechanisms of resistance and which drugs could be useful to overcome it is key. Among others, KRAS G12C (ON) tricomplex inhibitors and different combination therapy strategies are being analyzed in clinical trials. Another area of interest is the potential role of co-mutations in treatment selection, particularly immunotherapy. The best first-line strategy remains to be determined and, due to the heterogeneity of KRAS, is likely to be based on combination therapies.

## 1 Background

In recent years, there has been an enormous advance in the diagnosis and treatment of NSCLC patients, thanks to the discovery of different oncogenes amenable to targeted therapy such as Epidermal Growth Factor Receptor (EGFR), Anaplastic Lymphoma Kinase (ALK), ROS proto-oncogene 1 (ROS1), B-Raf proto-oncogene (BRAF), mesenchymal-epithelial transition factor (cMET), rearranged during transfection (RET) or neurotrophic tyrosine receptor (NTRK) (1), together with the development of immune checkpoint inhibitors (ICPI) either as monotherapy or in combination with chemotherapy, that have changed the management of patients with advanced disease and improved long-term survival (2). However, lung cancer remains one of the leading causes of cancer-related mortality, with nearly 1.8 million deaths worldwide in 2020 (3).

KRAS is the most common oncogenic mutation detected in patients with lung adenocarcinoma (LUAD) in the Western world, being found in approximately 20-25% of patients with NSCLC, most of them LUAD (4).

KRAS mutant(mt) NSCLC is a heterogeneous disease, which differs from other oncogene-driven tumors such as EGFR or ALK. This heterogeneity may be related to co-occurring genomic alterations, different KRAS mutations or tumor dependence/independence on KRAS, among others, that could condition intrinsic or acquired resistance to different treatments.

### 1.1 KRAS Biology and Mutations in Lung Cancer

The KRAS proto-oncogene encodes an intracellular guanine nucleotide-binding protein (G protein) belonging to the family of small GTPases. The structure of the KRAS protein consists of six beta chains and five alpha helices comprising a catalytic domain (G domain), which binds guanine nucleotides and activates signaling, and a C-terminal hypervariable region (HVR) that incorporates farnesyl or prenyl groups (post-transcriptional modifications) to drive the anchoring of KRAS to the membrane (5, 6). There are two isoforms of KRAS as a result of alternative splicing (KRAS4A and KRAS4B) with different posttranslational modifications and membrane localization. KRAS 4A might have a role in stress adaptability, such as hypoxia, and KRAS 4B might be overexpressed in stem cells (5, 7, 8).

Downstream signaling is regulated by the switch between the active state of guanosine triphosphate (GTP) and the inactive state of guanosine diphosphate (GDP) (6, 9). The RAS-GTP complex activates several downstream signaling effectors such as Raf-MEK-ERK, the phosphoinositide 3-kinase/protein kinase B/mechanistic target of rapamycin kinase (PI3K/AKT/mTOR), Ral guanine nucleotide dissociation stimulator (RALGDS-RalA/B pathways or the TIAM1-RAC1 pathway, which control multiple cellular functions including proliferation, apoptosis, metabolic changes, motility and survival (4, 6, 9, 10).

GDP-GTP exchange is regulated by additional proteins: Guanine nucleotide Exchange Factors (GEFs), such as Son-Of Sevenless (SOS), which decrease the affinity of RAS proteins for GDP and favor GTP binding, resulting in RAS activation, while GTPase activating proteins (GAPs), exemplified by neurofibromin (NF1), accelerate intrinsic GTPase activity to regulate RAS cycling. GEFs and GAPs bind to one or both of the binding pockets in the RAS (known as switch I and switch II regions) and these signaling cascades are triggered by the engagement of several receptor tyrosine kinases (RTKs) such as EGFR, human epidermal growth factor receptors 2-4 (HER2-4/ERBB2-4) or fibroblast growth factor receptor (FGFR) among others, which favor a constitutive activation of KRAS (5, 8, 11).

KRAS mutations are mostly point missense mutations occurring in exon 2 (codons 12 and 13) and, less frequently, in exon 3 (codon 61) of the G domain, impairing its GTP hydrolysis capacity and resulting in constitutive activation of KRAS proteins, promoting the GTP-bound active state (4, 10).

The frequency of KRAS mutations varies according to patient ethnicity, being more frequent in Western vs Asian populations (26% vs 11%), and more common in current or former smokers compared to non-smokers (30% vs 10%) (10, 11). It has also been observed that most KRAS mutations are clonal and appear early in carcinogenesis (12, 13). KRAS mutations are usually mutually exclusive of other predictive oncogenic mutations such as EGFR or ALK, although KRAS mutations may arise as a mechanism of resistance to targeted therapies (11, 13).

Although KRAS mutations have classically been defined as a negative prognostic factor, with more undifferentiated tumors having unfavorable survival and disease-free survival rates compared to KRAS wild-type (wt) tumors, the role of KRAS as a prognostic factor in NSCLC is not well established at this time due to heterogeneity among studies (14, 15).

### 1.2 KRAS Mutation Subtypes

The most frequent mutations in KRAS mt NSCLC are transversion mutations involving guanine to thymine or guanine to cytosine nucleotide changes, such as glycine 12 to cysteine (G12C) accounting for 41%, followed by glycine 12 to valine (G12V), both associated with a history of smoking, whereas transitions mutations, involving guanine to adenine nucleotide changes, such as glycine 12 to aspartic acid (G12D), are found mainly in never smokers (4).

It has been suggested that the type of point mutation may affect downstream signaling differently, which may translate into different clinical features and outcomes. G12C and G12V mutations are usually associated with the Ral A/B signaling pathway. However, KRAS G12D mutations preferentially activate PI3K/AKT and MEK signaling, and are often associated with non-smokers, especially KRAS G12D which is also associated with mucinous histology (4, 10).

There are differences in the patterns of metastasis depending on the KRAS mutation, with bone dissemination being more frequent in the KRAS G12C mutation, while the KRAS G12V mutation frequently presents with pericardial and pleural involvement (13, 16, 17).

### 1.3 KRAS-Dependency

Recent works have established two different groups of KRAS mt NSCLC: KRAS-dependent or KRAS-independent, according to their requirement for mutant KRAS to maintain tumor viability (4). KRAS-driven cells are associated with a well-differentiated epithelial phenotype, whereas non KRAS-driven cells correlate with an epithelial-mesenchymal transformation (EMT) phenotype (18–22).

### 1.4 Co-Mutational Status of KRAS

The co-mutational status of KRAS in NSCLC has been studied, showing that half (53%) of KRAS mt tumors had non-oncogenic co-mutations, the most frequent being TP53 (39%), serine/threonine kinase 11 (STK11) (20%), and kelch-like ECH-associated protein 1 (KEAP1) (13%), being probably clonal in nature and occurring early during oncogenesis (23–25). These findings correlate with those previously published by Skoulidis et al, who performed an integrative analysis of genomic, transcriptomic and proteomic data from early stage lung adenocarcinomas and metastatic tumors after progression to platinum, and identified three subtypes of KRAS mt NSCLC dominated respectively, by concurrent genetic events in STK11/LKB1 (the KL or subgroup 2), TP53 (KP, subgroup 3) and CDKN2A/B inactivation together with low expression of the transcription factor NKX2-1 (TTF1) (KC, subgroup 1), with relevant biological and therapeutic differences between the subgroups. KC tumors frequently had mucinous histology and suppressed mTORC1 signaling. KL tumors had high rates of KEAP1 mutational inactivation and expressed lower levels of immune markers, including PD-L1. Inactivation of the LKB1 gene may be driven primarily by genomic copy number suppression, inactivating mutation and down-regulation of its own expression, showing LKB1 protein depletion (26).

KP tumors showed higher levels of somatic mutations (although the smoking burden of the included patients was similar in all three subgroups), inflammatory markers, immune checkpoint effector molecules, and longer relapse-free survival (26, 27). Subsequently, Skoulidis analyzed the efficacy of antiPD-1 in advanced lines and observed higher responses in the KP versus KC subgroup (35.7% vs 7.4%), identifying STK11 as an antiPD-1 resistance mutation (28).

Consistent with this, Dong et al, observed that the TP53 mutation significantly increased PDL-1 expression and interferon gamma signature, more so in the TP53/KRAS mt subgroup, with increased antiPD-1 benefit in a small cohort of patients. However, heterogeneity has also been described in TP53-mutated LUAD, and there may be differences in response to ICPI depending on the type of TP53 mutation (29).

To date, clinical trials that have given approval to immune checkpoint inhibitor drugs targeting PD-1 or PD-L1 have not been designed or sufficiently powered to find differences between the molecular subgroups determined by Skoulidis based on KRAS co-mutational profiling (30–34).

### 1.5 KRAS Molecular Testing

KRAS can be performed as part of a multigene or Next-Generation Sequencing (NGS) panel or as a single-gene test. Single-gene tests, such as quantitative real-time PCR, droplet digital PCR, or pyrosequencing, can only detect prespecified mutations that are encoded in the molecular probe of a gene of interest, whereas NGS can detect multiple biomarkers from multiple genes related to carcinogenesis, with higher cost and more time (35, 36).

With the emergence of new predictive biomarkers for targeted therapies in NSCLC, NGS has become an essential genomic test in many institutions for clinical decision making, as it allows analyzing mutational hotspots in many oncogenes for different patients at the same time, which is crucial in patients with advanced NSCLC. Moreover, considering the heterogeneity of KRAS mt NSCLC, NGS would allow analyzing the presence of other co-mutations (36).

Circulating tumor DNA (ctDNA) liquid biopsy has been accepted as a noninvasive tool for diagnosis in patients without available or suitable tissue (37, 38). However, liquid biopsy has some limitations such as false positives of clonal hematopoiesis, i.e., accumulation of somatic mutations and clonal expansion of hematopoietic stem cells as a result of aging. Unlike other oncogenes, KRAS mutations arising from clonal hematopoiesis are rare false positives in liquid biopsy tests (13, 39).

To date, KRAS molecular testing is not indicated as a routine stand-alone assay as the sole determinant of targeted therapy (35). KRAS testing was performed to provide prognostic information or to rule out less common driver alterations (e.g., EGFR, ALK) mutually exclusive with KRAS mutations (38). However, this approach may soon change with the approval of KRAS-targeted drugs, which will require the determination of this biomarker.

## 2 KRAS and Immunotherapy

Initially, KRAS was associated with a favorable response to ICPI, as it was more frequently associated with smokers, high tumor mutational burden (TMB) and enhanced PD-L1, as well as high infiltration of immune cells (TILs) (13, 29, 30).

Exploratory, retrospective studies and meta-analyses have suggested that patients with KRAS mt may benefit from PD-1 blockade, without delving into the underlying mechanisms (31–34, 40).

Two meta-analyses that included three randomized phase II or III clinical trials examining Overall Survival (OS) in KRAS mt NSCLC ICPIs as second- or third-line therapy in mt KRAS NSCLC have shown contradictory results. The first one demonstrated an OS improvement compared to standard chemotherapy (HR = 0.64 [95% confidence interval, 0.43–0.96], *P* = 0.03) without significant OS benefit between ICPIs and chemotherapy in KRAS wt NSCLC (HR = 0.88 [95% confidence interval, 0.68–1.13], *P* = 0.30) (41). However, another meta-analysis concluded that there was not enough evidence to recommend KRAS mt alone as a predictive biomarker for ICPIs as no significant treatment interaction for KRAS mt (KRAS mt HR0.86 vs. KRAS wt HR, 0.65; *P* = 0.24) was found (10, 42).

The phase III KEYNOTE-042 trial that demonstrated an OS benefit of pembrolizumab in the first-line setting versus platinum-based chemotherapy in patients with advanced NSCLC with PD-L1 expression ≥ 1%, evaluated in an exploratory analysis, the association between KRAS status and efficacy to ICPI. KRAS status was determined by whole exome sequencing (WES) of tumor tissue in 301 patients with LUAD of the 1274 randomized participants with NSCLC, being 69/301 (22.9%) KRAS mt LUAD. The benefit of pembrolizumab versus chemotherapy was independent of KRAS mutational status in LUAD, although it was more pronounced in KRAS mt patients, with an Objective Response Rate (ORR) of 56.7% vs. 18% for LUAD patients with KRAS mt than those with KRAS wt (29.1% vs. 21%), a median Progression Free Survival (PFS) of 12 vs. 6 months (HR= 0. 51 [95% confidence interval, 0.29-0.87]) for LUAD patients with KRAS mt, compared to a PFS of 6 vs 6 months (HR=1.00 [95% confidence interval, 0.75-1.34]) in LUAD patients with KRAS wt and a median OS of 28 vs 11 months (HR=0.42 [95% confidence interval, 0.22-0.81]) in patients with KRAS mt compared to 15 vs 12 months (HR=0.86 [95% CI 0.63-1.18]) in LUAD patients with KRAS wt LUAD tumors. Notably, tumors with KRAS mt had increased expression of PD-L1 and TMB (32).

Recently, a retrospective study evaluating the association of KRAS mutational status with the benefit of antiPD-1 versus chemo-antiPD1 in patients with PD-L1 ≥ 50% has been published. Among 1127 patients with advanced LUAD and PD-L1 expression ≥ 50%, the prevalence of KRAS mt was 50%, similar to that published in other studies (13, 32, 34). Among patients with KRAS mt, OS did not differ between those treated with antiPD1 monotherapy and chemo-antiPD1 (mOS, 21.1 vs 20.0 months; *P* = .78). However, among patients with KRAS wt status, those treated with antiPD1 monotherapy had worse survival than those treated with chemo-antiPD1, although this difference was not statistically significant (median OS, 13.6 vs 19.3 months; *P* = 0.06). These results suggested that patients with KRAS wt NSCLC and PD-L1 expression ≥ 50%, treated with anti-PD-1 in monotherapy had worse survival than patients with mt KRAS NSCLC, while there was no difference in survival with chemo-antiPD1, suggesting that chemo-antiPD1 might be preferable in patients with KRAS wt and high PD-L1 expression (33).

It is now known that KRAS mt NSCLC is a heterogeneous disease, which differs from other oncogene-derived tumors, and that this heterogeneity may be related to concurrent genomic alterations such STK11 or TP53, different subtypes of KRAS or tumor dependence/independence on KRAS. The influence of these factors on the response to ICPI is being studied. As it was mentioned before, TP53/KRAS mt NSCLC tumors are related to an inflammatory microenvironment, enriched in TILs, have an increased presence of neoantigens and high PDL1 expression levels, whereas LKB1 inactivation in KRAS mt NSCLC tumors generally generates a suppressive immune microenvironment which could be linked to the lack of response to antiPD-1/PD-L1 blockade alone described in some studies (27–29). However, the value of these mutations in guiding ICPI for NSCLC patients is still uncertain.

## 3 KRAS and Targeted Therapy

### 3.1 Direct Targeting of KRAS G12C

#### 3.1.1 KRAS G12C (OFF) Inhibitors

KRAS proteins are small proteins with a relatively smooth molecular surface without readily accessible binding pockets, with a high affinity for GDP/GTP and complex downstream pathways (5, 43, 44). Therefore, direct targeting KRAS by small molecule inhibitor was a difficult approach until the discovery of a new pocket beneath the effector binding switch II region of KRAS glycine-to-cysteine amino acid substitutions at codon 12 (KRAS G12C), that has allowed the development of direct KRAS G12C inhibitors (45–47). Initially it was thought that mutation of KRAS led to constitutive activation in its GTP-bound state. However, KRAS G12C presents an intrinsic GTPase activity, not presented in other KRAS subtypes, of importance for the activity and efficacy of the direct KRAS G12C inhibitors (8, 46).

**Figure 1:**
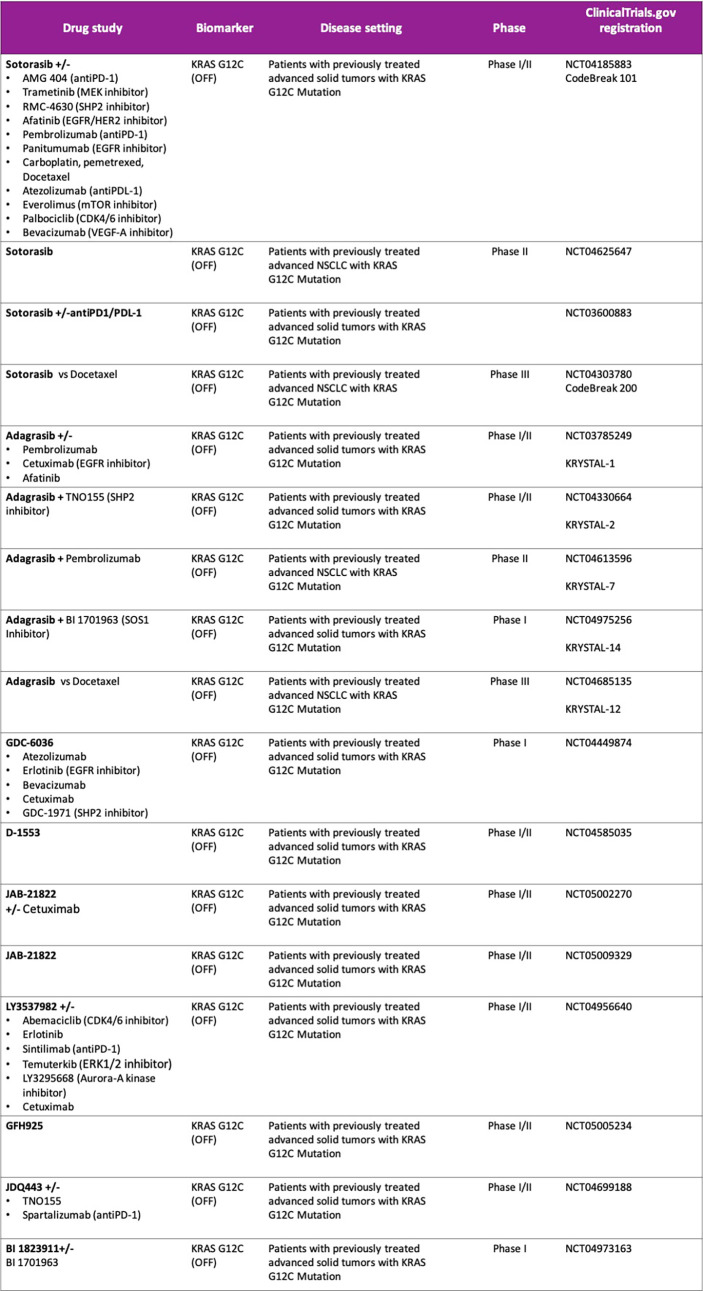
Ongoing studies with direct KRAS G12C inhibitors. Drug combination strategies.

Sotorasib (AMG 510) is an oral covalent KRAS G12C (OFF) inhibitor that irreversibly and selectively binds to the cysteine 12, next to pocket (P2) of the switch II region within KRAS mt, keeping it in the inactive GDP-bound state. It was evaluated in a phase I/II study (CodeBreak 100: NCT03600883) in pretreated KRAS G12C mt solid tumors (47, 48). At the 960 mg once-daily dose selected for phase II in patients with metastatic NSCLC (N = 126), the ORR was 37.1% and Disease Control Rate (DCR) was 80.6%. The median duration of response was 11.1 months, the median time to objective response was 1.4 months, with a median PFS 6.8 months (95% confidence interval, 5.1 to 8.2) and a median overall survival of 12.5 months (95% confidence interval, 10.0 to could not be evaluated). The activity of sotorasib was observed across a spectrum of prevalent co-occurring mutations such as STK11, KEAP1 or TP53 as well as different PD-L1 expression or TMB levels. However, these exploratory analyses were not statistically powered, subgroup sample sizes were small, and therefore future prospective studies are warranted to identify subgroups of patients who may benefit differently from sotorasib therapy. Treatment-related adverse events (TRAEs) occurred in 69.8% patients, including grade 3 events in 19.8%. Most common adverse events related to sotorasib were gastrointestinal side effects such as diarrhea (31.7%) and nausea (19%) as well as low-grade hepatic toxicity like alanine aminotransferase (ALT) and aspartate aminotransferase increase (AST) (each 15.1%). No fatal TRAEs were reported. Patients with active brain metastases were ineligible, so the efficacy of sotorasib in the treatment of patients with central nervous system metastases is unknown (48–50). Based on these results, Sotorasib was granted breakthrough designation by the U.S. Food and Drug Administration (FDA) for the treatment of adult patients with KRAS G12C mt locally advanced or metastatic NSCLC who have received at least one prior systemic therapy, becoming the first targeted therapy approved for advanced NSCLC KRAS G12C mt (50). The global phase III trial, CodeBreak 200 (NCT04303780), comparing sotorasib with docetaxel in patients with mt KRAS G12C NSCLC is ongoing, as well as different clinical trials are evaluating sotorasib in combination therapies (CodeBreaK101; NCT04185883) with the aim to identify patients who may benefit from sotorasib regimens in the context of first-line treatment (**Figure 1**).

Adagrasib (MRTX849) is another oral covalent KRASG12C inhibitor that irreversibly and selectively binds KRAS G12C in its inactive GDP-bound state. It was evaluated in a phase I/II study (KRYSTAL-1; NCT03785249) in pretreated patients with advanced solid tumors. At dose of 600 mg twice daily, of 51 evaluable patients with NSCLC, 45% had ORR and DCR was 96% with 8.2-month median duration of response. Regarding safety for all patients treated at the 600mg twice-daily dose (n = 110), grade 3 or 4 TRAEs occurred in 30% of patients; the most commonly reported (>5%) grade 3 or 4 TRAEs were fatigue (6%) and increased AST/ALT (5%). Two fatal TRAEs due to pneumonitis and cardiac failure were reported (51).

It has been also presented a preliminary analysis examining co-mutations with KRAS G12C. Patients who had KRAS G12C and STK11 co-mutations experienced an ORR of 64%, without apparent trends with KEAP1 or TP53, although the number of samples was small. Moreover, preclinical data and early phase studies indicate that adagrasib can penetrate the brain and cerebrospinal fluid (11, 51–53), although more data are needed to determine the brain activity of adagrasib.

A phase III trial (KRYSTAL-12) of adagrasib versus docetaxel for pretreated patients with KRASG12C-mutated NSCLC (NCT04685135) is ongoing and several combination strategies with adagrasib is under development (**Figure 1**).

(Similarities and differences among sotorasib and adagrasib (direct KRAS G12C inhibitors) are summarized in **Table 1**).

**Table 1:** Similarities and differences among sotorasib and adagrasib (direct KRAS G12C inhibitors).

Compound	Sotorasib (AMG 510)	Adagrasib (MRTX 849)
Mode of action and target	Covalent allosteric inhibitor KRAS G12C (OFF)	Covalent allosteric inhibitor KRAS G12C (OFF)
KRAS-GTP loading inhibition (IC_50 value_)	47.9 nM	89.9 nM
RP2D	960mg QD	600mg BID
Half-life	5.5 hours	24.7 hours
Study	Phase I/II study (CodeBreak 100; NCT03600883) in pretreated KRAS G12C mt solid tumors	Phase I/II study (KRYSTAL-1; NCT03785249) in pretreated KRAS G12C mt solid tumors
N	124 evaluable patients with advanced NSCLC KRAS G12C mt	51 evaluable patients
ORR	37.1%	45%
DCR	80.6%	96%
mPFS	6.8 months	—
mOS	12.5 months	—
Safety (TRAEs)	Any grade 69.8%	Any grade 85%
	G3 19.8%.	G3-4 30%
	Most common any grade TRAEs: Diarrhea (31.7%), nausea (19%) and ALT/AST increased (15.1%)	Most common G3-4 TRAEs: fatigue (6%) and AST/ALT (5%) increased.
Intracraneal activity	Patients with active brain metastases were ineligible	Adagrasib can penetrate the brain and cerebrospinal fluid (preclinical data) and has demonstrated antitumor activity against brain metastases (clinical data).

Other direct KRASG12C (OFF) inhibitors are in the early stages of clinical development as monotherapy and in combination with other therapies, such as GDC-6036, D-1553 JAB-21822, JDQ443 or LY3537982 (NCT04449874, NCT04585035, NCT05009329, NCT04699188, NCT04956640), that will be investigated alone or in combination with other study treatments (11, 13, 54, 55) (**Figure 1**).

On the other hand, LY3499446 and JNJ-74699157 (ARS 3248) were discontinued, the first one, due to safety issues.

#### 3.1.2 Tri-Complex Inhibitors of KRAS G12C (ON)

Novel second generation KRAS G12C inhibitors are under development in preclinical models, that consist of tri-complex inhibitors of the oncogenic GTP-bound form of KRAS G12C (ON) that overcome RTK-mediated escape mechanisms and lead to tumor regressions. The covalent tri-complex inhibitor of KRAS G12C (ON) exhibit a preclinical profile that is superior to the leading KRAS G12C (OFF) inhibitors in clinical development (13, 56). RMC-6291 is a first-in-class, potent, oral and selective tri-complex inhibitor of KRAS G12C (ON) and NRAS G12C (ON) that has demonstrated deep and sustained anti-tumor activity in preclinical lung and colorectal cancer models driven by a KRAS G12C mutation (57), and RMC-6236, another first-in-class, potent, oral RAS-selective tri-complex RAS^MULTI^(ON) inhibitor, which has demonstrated pronounced anti-tumor activity in preclinical models of human lung, colorectal and pancreatic cancers caused by multiple RAS variants including KRAS G12D and KRAS G12V and also in RAS-dependent wt tumors and RAS-mediated adaptive resistance tumors (13). Both drugs are pending of being tested in early phase trials.

#### 3.1.3 Intrinsic/Acquired Resistance Mechanisms to Direct KRAS G12C Inhibitors

A better understanding of the mechanisms of resistance to direct KRAS G12C inhibitors is crucial to guide combination strategies and the development of new drugs to improve outcomes for patients with KRAS mt NSCLC.

As previously mentioned, an independence of KRAS signaling and an epithelial-mesenchymal phenotype could lead to an intrinsic resistance to therapy based on direct KRAS G12C inhibitors (18–22).

A potential acquired resistance mechanism to direct KRAS G12C inhibitors is the reactivation following KRAS G12C inhibition driven by RTKs. The combination on RTKs inhibitors with direct KRAS G12C inhibitors as well as with Src homology phosphatase 2 (SHP2) inhibitors could reverse this reactivation (58–61).

A study of the possible mechanisms of acquired resistance to adagrasib from KRYSTAL-1 trial has recently been published. This study performed histologic and genomic analyses (NGS on tissue or ctDNA) developing a deep mutational scanning, and compared pretreatment samples of 38 patients (27 with NSCLC) who initially had stable disease for at least 12 weeks or an objective response to therapy followed by subsequent disease progression, with samples obtained after the development of resistance. 41% of patients had more than one concurrent potential resistance mechanism.

The most frequent on-target mechanisms to adagrasib included activating mutations in KRAS (G12D, G12V and G13D), Q61H), secondary KRAS mutations within the adagrasib-binding pocket (R68S, H95D/Q/R or Y96C) and high-level amplification of the KRAS G12C allele (62).

Recently, it has been suggested that the mechanisms of acquired resistance based on the presence of non-KRAS G12C mutations may be present at baseline, and selected by treatment with direct KRAS G12C inhibitors, becoming more prominent during the course of therapy, being potentially also involved in intrinsic resistance (63).

In relation to off-target mechanisms of resistance, the most frequent detected were MET amplification, activating mutations in NRAS, BRAF, MAP2K1, and RET; oncogenic fusions involving ALK, RET, BRAF, RAF1, and FGFR3; and inactivation mutations in NF1 and PTEN. Moreover, it was described histologic transformation to squamous-cell carcinoma in two patients with advanced NSCLC.

**Figure 2:**
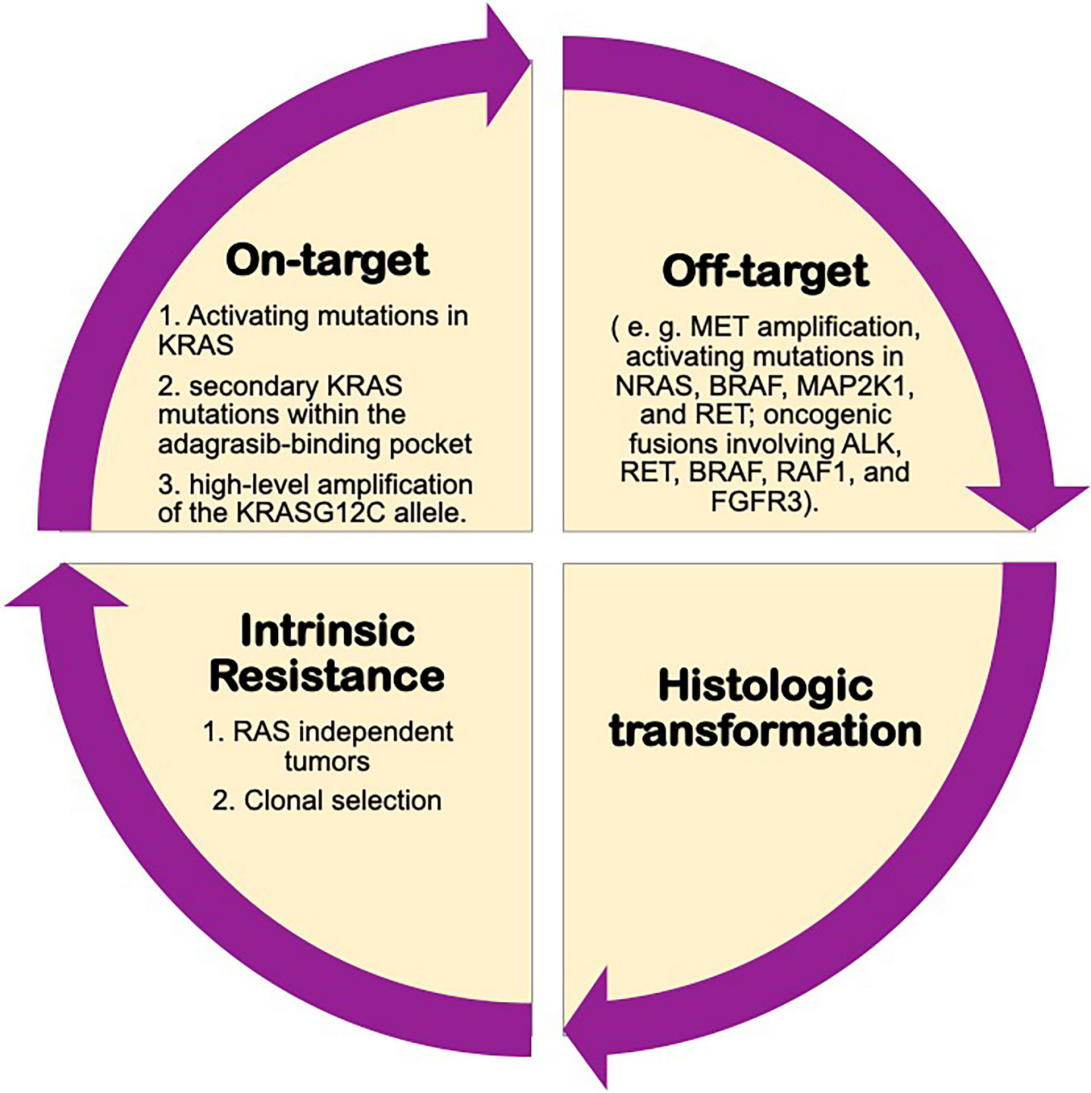
Possibly mechanisms of resistance to KRAS inhibitors.

In addition, these authors also performed *in vitro* experimental studies to compare these acquired resistance mechanisms with adagrasib and sotorasib, and it was seen that while R68S and Y96C mutations conferred resistance to both drugs, H95D/Q/R mutations do not confer *in vitro* resistance to sotorasib, as seen in patients treated with adagrasib (62). **Figure 2**.

Other authors studied *in vitro* model exposed Ba/F3 cells transduced with KRAS G12C, derived resistant cell lines to sotorasib or adagrasib, searching for secondary KRAS mutations, and identified Y96D and Y96S as resistant mutations to both drugs; while G13D, R68M, A59S and A59T were highly resistant to sotorasib but remained sensitive to adagrasib, and Q99L was resistant to adagrasib but sensitive to sotorasib. According to the different resistance mutations and their patterns of sensitivity to the different KRAS inhibitors, these authors proposed a possible treatment sequencing strategy (64).

### 3.2 Combination Strategies

**Figure 3:**
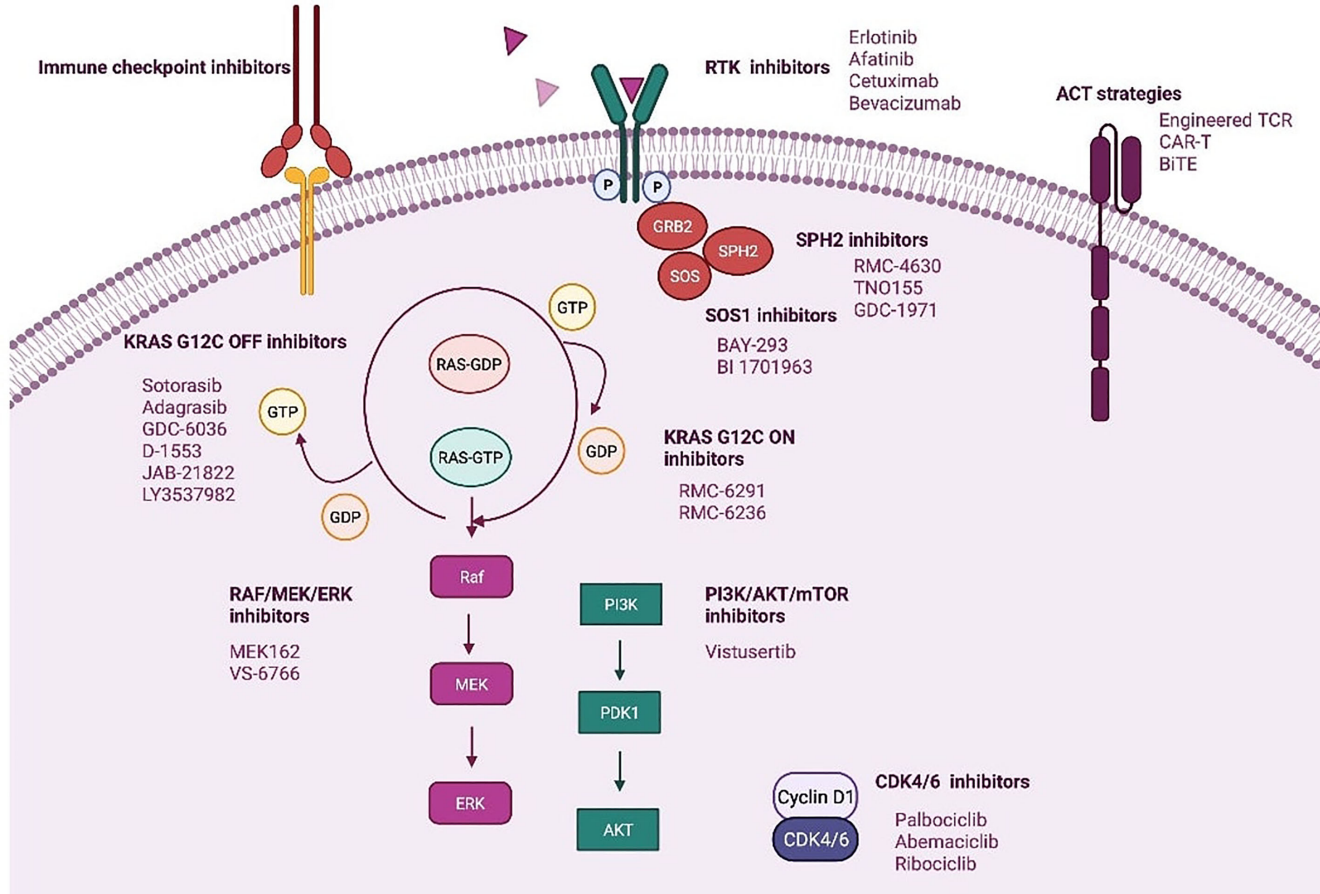
Novel strategies targeting KRAS mt NSCLC. Created in BioRender.com.

It seems that resistance to direct KRAS G12C inhibitors could involve diverse mechanisms that will imply a great challenge for the development of new targeted therapies and drug combination strategies (62). **Figure 3**.

#### 3.2.1 Combination of a KRASG12C Inhibitor With SHP2 Inhibitors

Src homology phosphatase 2 (SHP2) protein transduces signals from activated RTKs to downstream RAS pathways (65, 66). Recent studies have shown that SHP2 inhibition specifically suppresses the growth capacity of KRAS-mutant, but not wt NSCLC cells *in vitro*, which is promoted by TKI treatment (8, 11, 65–67).

There are several SHP2 inhibitors in development. RMC-4630 has shown clinical activity with a DCR of 67% for all KRAS mutations, and 75% for KRAS G12C mutations (phase I NCT03989115) (11, 58, 68, 69). TNO155 is a selective, allosteric, oral inhibitor of SHP2 and is being studied in a phase I trial in advanced solid tumors after disease progression following standard therapy (NCT03114319, NCT04330664) (68, 69).

SPH2 inhibition increases KRAS-GDP occupancy what it could increase the effect of direct KRAS G12C (OFF) inhibitors. This also has been demonstrated in preclinical studies of adagrasib combined with SHP2 inhibition (58, 66–69). Based on this, several ongoing clinical studies with both sotorasib and adagrasib are evaluating the combination of a KRAS G12C (OFF) inhibitor and a SHP2 inhibitor (NCT04330664, NCT04699188, NCT04185883).

SOS1 decreases the affinity of RAS proteins for GDP and favors GTP binding, leading to RAS activation. BI-3406 is a potent and selective inhibitor of the SOS1-KRAS interaction that attenuates reactivation by MEK inhibitors and enhances the sensitivity of KRAS-dependent cancers to MEK inhibition in preclinical models. Consequently, the combination of this new drug with a MEK inhibitor could be a good option for future research on KRAS-driven cancers (8, 11, 70).

Another inhibitor of the SOS1-KRAS interaction is BAY-293, which has demonstrated efficacy in KRAS-driven cancers in preclinical studies (71). BI-1701963 is a drug similar to BI-3406 that is being evaluated in a phase 1 trial in combination with a direct KRAS G12C inhibitor (OFF) (NCT04835714, NCT04975256) or trametinib (NCT04111458) in patients with advanced solid tumors with KRAS mutations.

#### 3.2.2 Combination KRAS Inhibition With RTKIs (Upstream Co-Inhibition)

The up-regulation activity of RTKs and, consequently, reactivation of RAS wt is an off-target mechanism of adaptive resistance to direct KRAS G12C (OFF) inhibitors. Therefore, vertical inhibition strategies are being developed to improve the clinical efficacy of KRAS G12C inhibitors (59, 72).

The KRYSTAL-1 study will evaluate adagrasib in combination with afatinib (an EGFR/HER2 inhibitor) or cetuximab (an EGFR monoclonal antibody) among other combinations, and CodeBreak 101 (NCT04185883) also includes an arm combining sotorasib with afatinib and another with panitumumab (an EGFR monoclonal antibody).

#### 3.2.3 Combination KRAS Inhibition With Downstream Co-Inhibition

The RAS-GTP complex activates several downstream signaling effectors, including the mitogen-activated protein kinase (MAP-K)/ERK and PI3K/AKT/mTORC1 pathways. Over the past decades, several attempts have been made to block these signaling pathways with disappointing results (8, 73).

In contrast to the first generation of RAF inhibitors, which failed due to activation of the RAF/MEK/ERK pathway in BRAF-like tumor cells, pan-RAF inhibitors with a more effective RAS pathway blocking profile are being developed (74, 75). Another potential strategy involves the combination of MEK inhibitors with direct KRAS G12C inhibitors (OFF) (NCT04185883). VS-6766 is a dual RAF/MEK inhibitor that blocks both the kinase activity of MEK and the ability of RAF to phosphorylate MEK (75, 76). The use of VS-6766 in combination with defactinib, an FAK inhibitor, is being investigated in patients with advanced KRAS mt solid tumors (NCT03875820).

The PI3K/AKT/mTORC1 pathway is not dependent on RAS alone for activation (13, 61, 72). In this setting, combination therapies including a direct KRAS G12C inhibitor and PI3K inhibitor could synergistically increase response (77).

Other therapeutic agents being studied in combination with direct KRAS (OFF) inhibitors include cyclin-dependent kinase 4/6 inhibitors (13).

#### 3.2.4 KRAS G12C (OFF) Inhibitors in Combination With ICPI

Sotorasib in combination with antiPD-1 has demonstrated complete responses in immunocompetent mice with patient-derived xenografts, and induced in these mice, a durable immune response with increased TILS and antigen presenting cells, greater benefit than that obtained with each agent in monotherapy (11, 43). On the other hand, adagrasib was shown to modulate factors involved in antigen presentation or an immunosuppressive tumor microenvironment in a panel of human xenograft models. Adagrasib was also shown in mice to decrease myeloid-derived immunosuppressive suppressor cells and increase M1-polarized macrophages, dendritic cells, and CD4+ and CD8+ T cells when administered as a single agent, whereas when administered in combination with anti-PD-1 therapy it leads to durable complete regressions through an immune-mediated antitumor response (78).

Early phase clinical trials are evaluating the combination of ICPI with the KRAS G12C inhibitor adagrasib or sotorasib (NCT03785249, NCT04185883, NCT03600883, NCT04613596) (43, 77).

It represents an attractive strategy for those KRAS subgroups less likely to respond to anti-PD1 monotherapy, such as the STK11/KRAS co-mutated subgroup.

The use of combination regimens of IPCI with TKIs has resulted in excess toxicities without additional efficacy in metastatic NSCLC with actionable driver mutations such as EGFR or ALK (79). To date, no increase in grade 3 or higher toxicities, such as interstitial lung disease or liver toxicity, has been reported with sotorasib or adagrasib in phase I/II clinical trials (48–51). However, the impact of KRAS G12C inhibitors on toxicities arising from prior ICPI use is not well understood and remains an important question, as most patients eligible for KRAS G12C inhibitors will have been previously exposed to ICPIs. Recently, a case of severe immune-related hepatitis likely triggered by sotorasib has been reported in a patient with KRAS G12C mt NSCLC who had been previously treated with antiPD-1 (80).

#### 3.2.5 Other Strategies

KRAS-targeted degradation might be an important therapeutic approach to KRAS mt tumors regardless of KRAS subtype.

PROteolysis TArgeting Chimeras (PROTACs) or small molecule degrader technology are novel compounds design to induce targeted protein ubiquitination and proteasomal degradation by the cereblon E3 ligase complex (81). These bifunctional molecules simultaneously engage a target protein and an E3 ligase, forming a ternary complex, which allows the E3 ligase to ubiquitinate the target protein at proximal lysine residues that is recognized and degraded by the 26S proteasome (82). Initially, PROTACs targeting KRAS G12C did not degrade endogenous KRAS (83). However, the emergence of covalent inhibitors to target KRAS has enabled the development of PROTACs capable of inducing endogenous KRASG12C degradation in cancer cells, such as LC-2 that couples the covalent KRASG12C inhibitor adagrasib to the von Hippel–Lindau (VHL) ligand (82).

## 4 Vaccines and Adoptive Cell Therapy

KRAS mutations are cancer-specific and do not exist in normal tissues (84), constituting mostly driver mutations that are ideal vaccine and ACT targets due to their clonal nature. More than 20 years ago it was shown that KRAS mt protein peptides were immunogenic, could be presented by the major histocompatibility complex (MHC) and undergo antigen recognition by T-cell receptors (TCRs) (85, 86).

### 4.1 Cancer Vaccines

Several KRAS vaccines are being evaluated in early phase clinical trials, alone or in combination with antiPD-1 therapy.

V941 is an mRNA-based cancer vaccine formulated with lipid nanoparticles that targets four of the most prevalent KRAS mutations (G12D, G12V, G13D and G12C). V941 induces cytotoxic T lymphocyte (CTL)- and memory T cell-dependent immune responses that specifically target and destroy tumor cells harboring these specific KRAS mutations (58). It is being evaluated in an ongoing Phase I study (NCT03948763) in patients with advanced or metastatic NSCLC, colorectal or pancreatic adenocarcinoma, alone or in combination with pembrolizumab. In part 2 of the study, patients with HLA-A*1101 and/or HLA-C*0802, most likely to respond to pembrolizumab, will be selected.

### 4.2 Adoptive Cell Therapy (ACT) and Bispecific T-cell Engager (BiTE)

ACT involves the use of tumor-reactive T cells expanded ex vivo and administered to a recipient after having undergone preparative lymphodepletion. It is based on the use of genetically modified T cells driven to the cancer cells through the introduction of a synthetic T Cell Receptor (TCR) or a Chimeric Antigen Receptor (CAR) (87, 88).

Tran et al. identified for the first-time polyclonal reactivity of CD8+ TILs against KRAS G12D in TILs from a patient with colorectal carcinoma carrying the G12D mutation and HLA- C*08:02, after infusion of expanded TILs, which achieved objective tumor regression in multiple pulmonary metastases (89).

The use of cloned TCR technology might be more appropriate for direct targeting of KRAS mt antigens, as they are present on the inner leaflet of the cell membrane. Multiple cloned TCRs that recognize specific KRAS subtypes are being developed. Autologous T cells transduced with murine KRAS G12D-specific TCR and KRAS G12V-specific TCR for HLA-A*11:01 in patients with advanced solid tumors are currently being evaluated in phase I/II clinical trials (NCT03745326, NCT03190941 respectively).

Among the limitations of engineered TCR are the restriction of this treatment to patients with a specific HLA subtype and, on the other hand the potential mechanisms of resistance such as loss of antigen, HLA or interferon gamma signaling (87, 90).

Both CAR-T and BiTE are HLA-independent therapies that could overcome the limitations of engineered TCRs related to patient selection based on specific HLA subtype, as well as the mechanism of resistance secondary to HLA loss. However, the intracellular nature of KRAS makes direct antigen binding difficult. Specific driver mutations in NSCLC may be associated with high levels of expression of multiple tumor surface antigens potentially amenable to targeting CAR-T and BiTE strategies (90).

KRAS mt NSCLC has previously been associated with increased mesothelin expression and an indirect approach is the development of CAR-Ts directed against mesothelin (91, 92).

The need to perform leukopheresis and lymphodepletion, which involves hospitalization, the high complexity of the manufacturing components, as well as the potential serious side effects arising from this therapy such as cytokine release syndrome (CRS), immune effector cell-associated neurotoxicity syndrome (ICANS) or infections secondary to prolonged aplasia are the powerful challenges posed by these therapies (90).

## 5 Conclusions

Despite multiple efforts to develop therapies directed against RAS or its signaling pathways, the fact is that, to date, first-line treatment in advanced mt KRAS NSCLC does not differ from NSCLC without actionable driver genomic alterations. Recent advances in the understanding of the structure of mutant KRAS have led to the development of new allele-specific inhibitors that have shown promising efficacy in pretreated advanced KRAS mt G12C NSCLC patients in phase I/II clinical trials.

These direct KRAS G12C inhibitors alone or in combination with therapies that target RAS-activating or RAS effector pathways as well as ICPI are being evaluated in phase III and phase I/II clinical trials respectively, with the aim of providing better outcomes.

## Author Contributions

Construction of review was performed by ECF and PG. Review was performed by MOG, AGR and YL. All authors contributed to the article and approved the submitted version.

## Conflict of Interest

PG declares personal financial interests as advisor for AbbVie, AstraZeneca, Blueprint Medicines, Boehringer Ingelheim, Bristol, Gilead, Guardant Health, Janssen, Lilly, MSD, Novartis, Pfizer, Roche, Rovi, Sysmex and Takeda.

The remaining authors declare that the research was conducted in the absence of any commercial or financial relationships that could be construed as a potential conflict of interest.

## Publisher’s Note

All claims expressed in this article are solely those of the authors and do not necessarily represent those of their affiliated organizations, or those of the publisher, the editors and the reviewers. Any product that may be evaluated in this article, or claim that may be made by its manufacturer, is not guaranteed or endorsed by the publisher.
